# Alterations in vitamin D status and anti-microbial peptide levels in patients in the intensive care unit with sepsis

**DOI:** 10.1186/1479-5876-7-28

**Published:** 2009-04-23

**Authors:** Leo Jeng, Alexandra V Yamshchikov, Suzanne E Judd, Henry M Blumberg, Gregory S Martin, Thomas R Ziegler, Vin Tangpricha

**Affiliations:** 1 Division of Endocrinology, Diabetes & Lipids, Department of Medicine, Emory University School of Medicine, Atlanta, GA, USA; 2 Division of Infectious Diseases, Department of Medicine, Emory University School of Medicine, Atlanta, GA, USA; 3 Nutrition and Health Sciences Program, Graduate Division of Biological and Biomedical Sciences, Emory University, Atlanta, GA, USA; 4 Division of Pulmonary and Critical Care Medicine, Department of Medicine, Emory University School of Medicine, Atlanta, GA, USA; 5 Center for Clinical and Molecular Nutrition, Department of Medicine, Emory University School of Medicine, Atlanta, GA, USA; 6 Atlanta VA Medical Center, Decatur, Georgia

## Abstract

**Background:**

Vitamin D insufficiency is common in hospitalized patients. Recent evidence suggests that vitamin D may enhance the innate immune response by induction of cathelicidin (LL-37), an endogenous antimicrobial peptide produced by macrophages and neutrophils. Thus, the relationship between vitamin D status and LL-37 production may be of importance for host immunity, but little data is available on this subject, especially in the setting of human sepsis syndrome and other critical illness.

**Methods:**

Plasma concentrations of 25-hydroxyvitamin D (25(OH)D), vitamin D binding protein (DBP) and LL-37 in critically ill adult subjects admitted to intensive care units (ICUs) with sepsis and without sepsis were compared to healthy controls.

**Results:**

Critically ill subjects had significantly lower plasma 25(OH)D concentrations compared to healthy controls. Mean plasma LL-37 levels were significantly lower in critically ill subjects compared to healthy controls. Vitamin D binding protein levels in plasma were significantly lower in critically ill subjects with sepsis compared to critically ill subjects without sepsis. There was a significant positive association between circulating 25(OH)D and LL-37 levels.

**Conclusion:**

This study demonstrates an association between critical illness and lower 25(OH)D and DBP levels in critically ill patients as compared to healthy controls. It also establishes a positive association between vitamin D status and plasma LL-37, which suggests that systemic LL-37 levels may be regulated by vitamin D status. Optimal vitamin D status may be important for innate immunity especially in the setting of sepsis. Further invention studies to examine this association are warranted.

## Introduction

Vitamin D is a pro-hormone important for serum calcium and phosphorus homeostasis for proper neuromuscular function and optimal skeletal health. Vitamin D can be obtained from the diet or made in the skin after exposure to ultraviolet B radiation from the sun. Vitamin D is then converted to its major circulating form, 25-hydroxyvitamin D (25(OH)D), by the liver and to its hormonally active form, 1,25-dihydroxyvitamin D (1,25(OH)_2_D), by the kidney to increase the efficiency of intestinal absorption of calcium as its classic function.

Recent studies suggest that vitamin D may have other actions outside of its classic functions related to bone and calcium homeostasis [1]. Cells of the innate and adaptive immune system including macrophages, lymphocytes and dendritic cells express the vitamin D receptor (VDR) and respond to stimulation by 1,25(OH)_2_D [2,3]. Cathelicidin (known as LL-37; which is cleaved from its precursor hCAP18) is an endogenous antimicrobial peptide (AMP) active against a broad spectrum of infectious agents including gram negative and positive bacteria, fungi and mycobacteria [4]. In vitro, 1,25(OH)_2_D_3 _treatment of cultured macrophages infected with *Myobacterium tuberculosis *(*M. tb*) leads to enhanced expression of cathelicidin [3]. Cathelicidin is highly expressed at barrier sites including respiratory and colonic epithelium, saliva, and skin and thus provides an important first line defense mechanism for the innate immune system to respond to infectious insults. Stimulated macrophages cultured in vitamin D deficient sera are unable to up-regulate LL-37 and effectively kill *M. tb *[3]. The addition of 25(OH)D to the media up-regulated production of LL-37 and restored effective killing of *M. tb*, suggesting that vitamin D has an important role in the production of anti-microbial peptides important for innate immunity [3].

Patients with severe infections as in sepsis have a high prevalence of vitamin D deficiency [5,6] and high mortality rates [7]. Furthermore, epidemiologic findings have implicated vitamin D insufficiency as a risk factor for sepsis [8]. The role of vitamin D treatment in sepsis syndrome has been evaluated in animal models of sepsis where 1,25(OH)_2_D_3 _administration was associated with improved blood coagulation parameters in sepsis associated disseminated intravascular coagulation (DIC) [9,10]. Vitamin D treatment in vitro has also been demonstrated to modulate levels of systemic inflammatory cytokines such as TNF-α and IL-6 [11,12], as well as to inhibit LPS-induced activation and vasodilation [13] of the vascular endothelium. These effector functions of vitamin D may be of importance in the pathogenesis of sepsis and sepsis-related DIC, especially when considered together with the potential for vitamin D to enhance anti-microbial peptide production. Furthermore, serum levels of vitamin D binding protein (DBP), the major carrier protein of vitamin D, are decreased in the setting of sepsis leading to lowered levels of 25(OH)D [14].

The role of vitamin D in sepsis syndrome has not been fully evaluated in humans. Therefore, we performed a cross-sectional study of vitamin D status including plasma levels of 25(OH)D and vitamin D binding protein (DBP) and their relationship to systemic LL-37 levels in a group of critically ill patients including those with and without sepsis.

## Methods

### Study Sample and Subjects

This study was approved by the Emory University Institutional Review Board. Samples were taken from three patient populations: Group 1 consisted of 24 critically ill subjects in the intensive care unit (ICU) patients diagnosed with sepsis (as defined by the American College of Chest Physicians (ACCP) and Society of Critical Care Medicine (SCCM) consensus panel in 2001 [15]; Group 2 consisted of 25 ICU subjects without the diagnoses of sepsis, and Group 3 consisted of 21 healthy non-hospitalized controls. Samples were collected between January of 1999 and May of 2006. Group 1 samples were drawn within 2 days of severe sepsis onset and were drawn from the medical intensive care unit between June 2004 and February 2006. Group 2 samples were drawn during the subject's hospital day, which was a mean of 12.8 days.

Critically ill subjects (groups 1 and 2) were characterized by sex, race, Acute Physiology and Chronic Health Evaluation II (APACHEII) and sequential organ failure assessment (SOFA) scores (for ICU patients) and whether they were diagnosed with cardiovascular disease (ischemic heart disease or congestive heart failure), liver disease, chronic renal failure, diabetes, HIV, or cancer. Subjects also had baseline laboratory tests performed by standard hospital laboratory methods including albumin, prothrombin time (PT), partial thromboplastin time (PTT), INR (International Normalized Ratio), Alanine aminotranferease (ALT), aspartate aminotransferase (AST), blood urea nitrogen (BUN), creatinine (Cr), hemoglobin, and white blood cell count. Diagnoses and laboratory data were obtained from discharge summaries and computer databases.

Healthy control subjects (group 3) were adults without known acute or chronic diseases, no hospitalizations for any illness previous 12 months, not taking any medications or vitamin supplements. They were screened for inclusion by a physician (TRZ) in General Clinical Research Clinic (GCRC) setting to confirm normal history and physical exam and had normal complete blood count, chemistry profile and urinalysis, which were tested within 2 weeks of screening.

### Plasma collection and 25(OH)D, vitamin D binding protein and LL-37 concentrations

Plasma was collected after informed consent was obtained from either the donor or from their family. Plasma samples were obtained in EDTA tubes and centrifuged for 20 minutes at 1100 – 1300 rpm. The plasma was stored at -80°C prior to analysis. Plasma levels of 25(OH)D and vitamin D binding protein (DBP) were assessed using ELISA (IDS, LTD, Fountain Hills, Arizona & Alpco, Salem, New Hampshire, respectively). Plasma levels of LL-37 were determined by ELISA (Hycult biotechnology, Uden, The Netherlands). Protocols for each assay were per the manufacturer's product manuals. Samples for 25(OH)D and DBP were tested in duplicates and LL-37 in single measurements. The intra-assay CV for 25(OH)D, DBP and LL-37 were <8%, <5% and <10%, respectively. The inter-assay CV for 25(OH)D, DBP and LL-37 were <10%, <13% and <10%, respectively. Vitamin D insufficiency was defined as a 25(OH)D concentration < 30 ng/mL.

### Statistical analysis

We used GraphPad Prism version 4.0 software (La Jolla, CA) to compare means of each of the lab values across the three groups of patients (critical ill subjects with and without sepsis and healthy controls). Multiple regression was used to calculate adjusted least squares means using PROC GLM in SAS 9.1 (SAS Institute Cary, NC) between the three subject populations. Fisher's exact test was used to examine differences in race across the three patient groups. Similarly, patients with darker skin pigmentation are at increased risk for vitamin D deficiency due to the absorption of solar radiation between 290 to 700 nm by melanin [15], therefore, we controlled for racial differences in all multivariate models. We also examined age and BMI as potential covariates but they were not significantly associated with LL-37. One patient sample was dropped from the analysis because the LL-37 level was more than three standard deviations from the mean. Data are presented as means ± SD.

## Results

### Patient demographics

The three groups of subjects (critically ill subjects with and without sepsis and healthy controls) were similar in distribution of gender and age. Co-morbid conditions that existed prior to hospital admission including underlying liver disease, diabetes, cardiovascular disease, and malignancy were similar among the three groups. Four subjects with HIV were present in the critically ill group with sepsis and not present in the other two groups. There were significantly more patients of black or African-American race in critically ill group with sepsis compared to the critically ill group without sepsis but was similar to the healthy controls (Table 1).

**Table 1:** Baseline Demographics of Patient Groups

	**ICU** **Sepsis**	**ICU** **Controls**	**Healthy** **Controls**	**ANOVA** **P-value**
Number of Subjects	24	25	21	

Age, mean (SD)	54.0 (17.1)	56.1 (15.9)	46.5 (6.1)	0.13

Male Gender, n (%)	15 (58)	13 (52)	17 (80)	0.49

Black or African American race, n (%)	22 (92)†	14 (56)	15 (71)	0.02

White race, n (%)	2 (8)	11 (44)	5 (29)	0.02

HIV infected, n (%)	4 (17)	0	0	0.02

Diabetes, n (%)	9 (38)	6 (24)	2 (10)	0.09

Cardiovascular disease, n (%)	14 (58)	17 (68)	8 (38)	0.12

Malignancy, n (%)	2 (8)	0	1 (5)	0.35

APACHE Score, mean (SD)	25.7 (7.4)	11.8 (5.4)	N/A	<0.0001

SOFA, mean (SD)	11.9 (4.0)	5.3 (3.2)	N/A	<0.0001

Albumin, mean (SD) mg/dL	2.0 (2.6)‡	2.1 (0.7)#	4.0 (0.5)	<0.0001

Prothrombin Time (PT) mean (SD)*	16.7 (2.9)	15.3 (2.8)	16.8 (3.9)	0.98

Partial thromboplastin time (PTT) mean (SD)*	48.8 (37.0)	35.1 (5.3)	34.8 (1.7)	0.05

INR, mean (SD)	1.6 (0.4)†‡	1.2 (0.3)	1.3 (0.4)	0.0009

AST, mean (SD), units/L	87.0 (131)	49.9 (48)	33.0 (26)	0.09

ALT, mean (SD), units/L	43.6 (44)	51.3 (75)	25.4 (17)	0.24

BUN, mean (SD), mg/dL	52.4 (28) †‡	27.8 (17)#	12.6 (5.1)	<0.0001

Creatinine, mean (SD), mg/dL	4.0 (3.2) †‡	1.1 (0.7)	1.0 (1.1)	<0.0001

Hemoglobin, mean (SD) g/dL	9.8 (2.0)‡	10.6 (1.2)#	13.8 (1.1)	<0.0001

White Blood Count (cells/μL)	17.3 (13)‡	15.2 (7.1)#	6.5 (1.9)	<0.0002

* in seconds

Tukey-Kramer Multiple Comparisons Test

† P < 0.05, ICU Sepsis vs ICU Controls

‡ P < 0.05, ICU Sepsis vs Healthy Controls

# P < 0.05, ICU Control vs Healthy Controls

Critically ill subjects with sepsis exhibited higher severity of illness scores (APACHEII and SOFA) than critically ill subjects without sepsis. APACHEII and SOFA scores were not applied to healthy controls. In addition, critically ill subjects with sepsis had significantly more derangements in metabolic and hematologic parameters than ICU control subjects and healthy subjects (Table 1). For example, critically ill subjects with sepsis had significantly higher INR, BUN and creatinine measurements, signifying increased incidence of multiple organ dysfunction and DIC in the setting of sepsis syndrome. Each of the two critically ill groups also demonstrated a significantly higher prevalence of anemia and leukocytosis than healthy controls, as expected in the setting of illness requiring intensive care. Both critically ill groups also had significantly lower serum albumin, indicating that the two groups had higher disease severity and possibly more nutritionally impaired than healthy controls (Table 1).

### Plasma 25-hydroxyvitamin D, Vitamin D Binding Protein, and LL-37 Concentrations in Critically Ill Subjects With and Without Sepsis and Healthy Subjects

**Figure 1:**
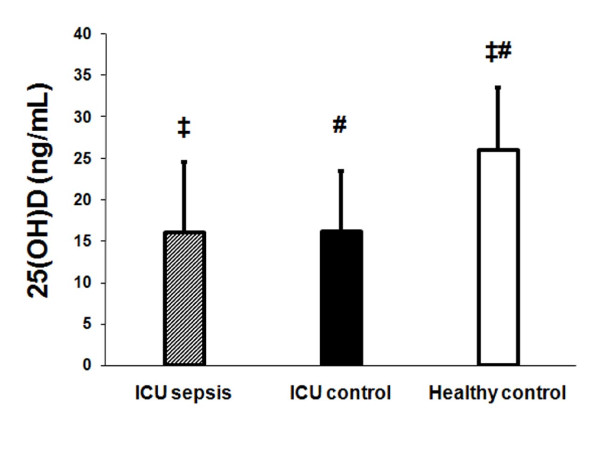
**Vitamin D status in critically ill subjects with sepsis, critically ill subjects without sepsis and healthy subjects**. Plasma 25-hydroxyvitamin D levels in critically ill subjects with sepsis (hatched bar) and in critically ill control subjects without sepsis (dark bar) were significantly lower than healthy controls (white bar) (ANOVA, p < 0.0001). 25-hydroxyvitamin D concentrations were adjusted for race. ‡ p < 0.001, critically ill sepsis subjects compared to healthy controls. # p < 0.01, critically ill control subjects compared to healthy controls.

Vitamin D status differed in the critically ill subjects with sepsis, critically ill subjects without sepsis and healthy controls (p < 0.0001, ANOVA). Race adjusted 25(OH)D concentrations demonstrated no significant differences in 25(OH)D between the two critically ill groups. However, the mean race adjusted 25(OH)D level in the two critically ill groups (16.0 ± 8.5 and 16.2 ± 7.2 ng/mL, sepsis and non-sepsis respectively) was significantly lower than healthy controls (26.0 ± 7.6 ng/mL) (Figure 1). The prevalence of vitamin D insufficiency (defined as 25(OH)D < 30 ng/ml) in critically ill subjects with sepsis was 100% (24/24) and 92% (23/25) in critically ill subjects without sepsis. In contrast, the prevalence of vitamin D insufficiency in the healthy controls was 66.5% (14/20) (p = 0.003); there was no significant difference in the prevalence of vitamin D insufficiency between the two critically ill groups (p = 0.15).

**Figure 2:**
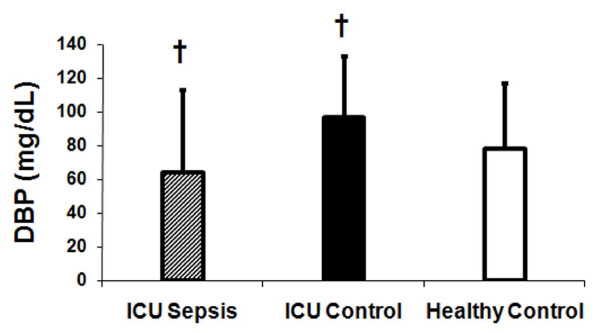
**Plasma vitamin D binding protein in critically ill subjects with sepsis, critically ill subjects without sepsis and healthy subjects**. Plasma vitamin D binding protein concentrations were significantly lower in critically ill subjects with sepsis (hatched bar) compared to critically ill control subjects (dark bar) (white bar) (ANOVA, p = 0.014). † p = < 0.05, critically ill sepsis subjects compared to critically ill control subjects.

**Figure 3:**
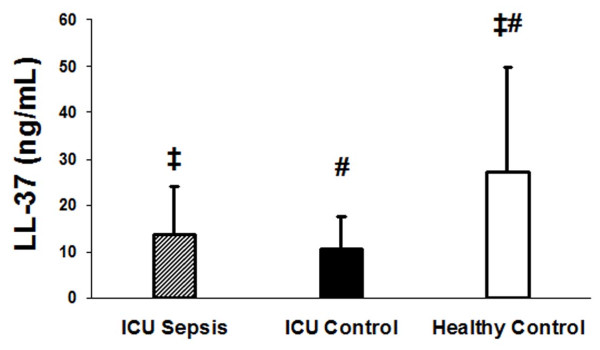
**Anti-microbial peptide cathelicidin (LL-37) in critically ill subjects with sepsis, critically ill subjects without sepsis and healthy subjects**. Plasma LL-37 levels were significantly lower in the two critical ill groups (with sepsis, hatched bar and without sepsis, dark bar) compared to the healthy controls subjects (white bar) (ANOVA, p= 0.002). There was no statistically significant difference between LL-37 levels in the two critically ill groups. ‡ p < 0.001, critically ill sepsis subjects compared to healthy controls. # p < 0.001, critically ill control subjects compared to healthy controls.

There was a statistically significant difference in plasma vitamin D binding protein between the three groups of subjects (ANOVA, p = 0.014) (Figure 2). Subjects with sepsis had significantly lower DBP concentrations compared to subjects without sepsis (Figure 2). Race was not associated with DBP levels. Cathelicidin (LL-37) concentrations differed in the three groups (p = 0.002, ANOVA). Both groups of critically ill subjects had similar plasma LL-37 concentrations (Figure 3, 13.7 ± 2.1 ng/mL vs. 10.6 ± 1.4 ng/mL; P = 0.59). However, mean plasma LL-37 levels in healthy controls (27.2 ± 4.9 ng/mL) were significantly higher than compared to the critically ill groups (p < 0.001, Tukey-Kramer for both comparisons) (Figure 3). While adiposity has been associated with 25(OH)D levels, we tested the association of BMI with LL-37 and showed no statistical significant relationship (p = 0.20). Therefore, BMI was not used in any of our models. Plasma LL-37 levels also were not significantly associated with age or race.

### Vitamin D status and relationship to LL-37 levels

**Figure 4:**
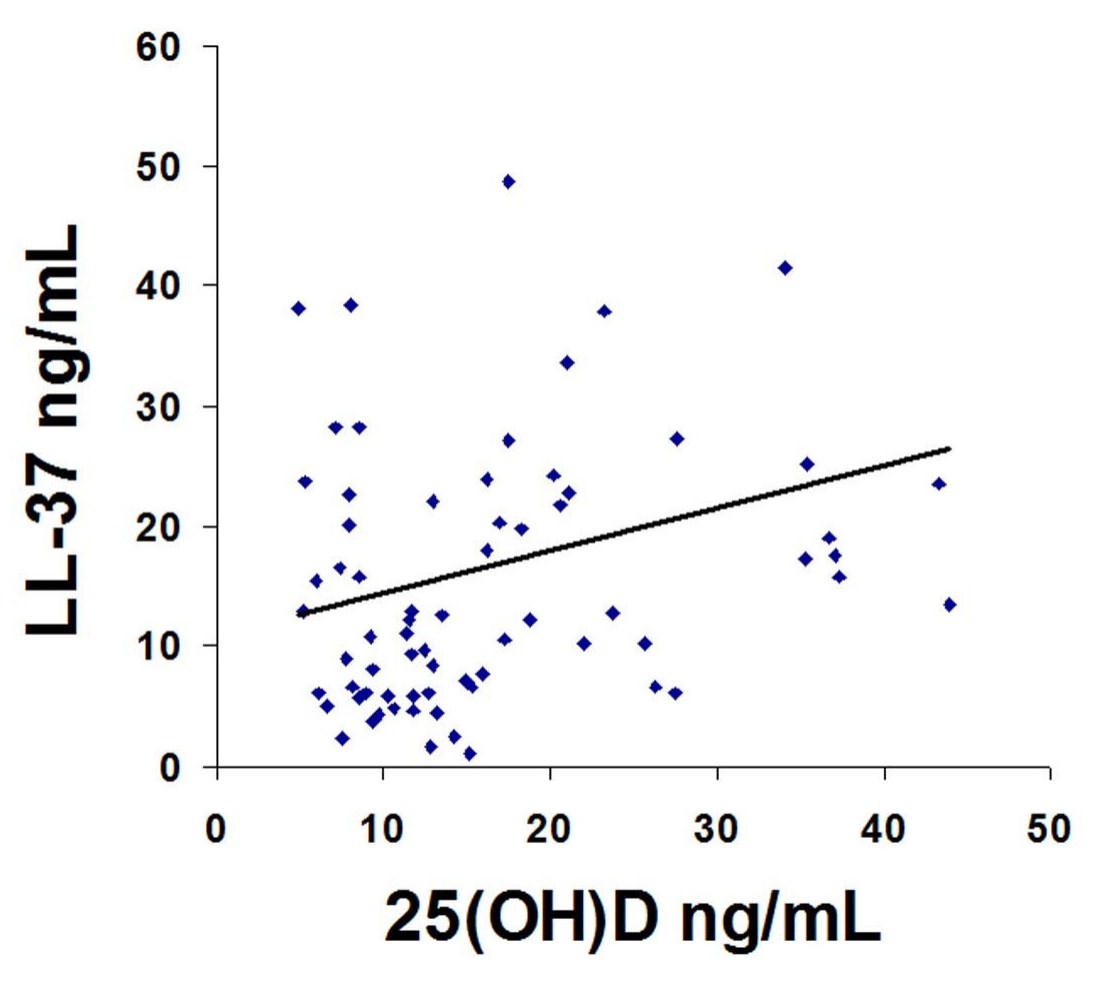
**Relationship between plasma 25-hydroxyvitamin D and cathelicidin (LL-37) in critically ill subjects with sepsis, critically ill subjects without sepsis and healthy subjects**. The was a positive relationship between plasma 25-hydroxyvitamin D (25(OH)D) and systemic LL-37 levels in all three subject groups (critically ill subjects with sepsis, critically ill without sepsis and healthy controls). This remained significant after adjustment for differences in race and patient population (R^2 ^= 0.21, P = 0.05).

To determine whether there was an association between 25(OH)D and LL-37, we plotted LL-37 levels against 25(OH)D in all subjects in this study. We found a positive linear correlation between 25(OH)D and LL-37 (R = 0.2385, p = 0.049), which remained statistically significant after controlling for race (Figure 4, R = 0.28, p = .05). When we reran our linear regression and included the group category as both a covariate and interaction term with 25(OH)D, the interaction was not statistically significant (p = 0.72). However, group was a significant predictor and increased the r-squared of the model from 0.05 to 0.21. The p-value for 25(OH)D remained at 0.05.

## Discussion

We have demonstrated that vitamin D insufficiency is highly prevalent in all three populations. Even in healthy controls, over sixty percent were found to be vitamin D insufficient. However, the prevalence of vitamin D insufficiency is even higher in subjects admitted to the intensive care unit with critical illness. We also demonstrate that vitamin D binding protein levels are significantly lower in critically ill subjects with sepsis compared to critically ill subjects without sepsis and healthy controls. When we examined plasma levels of the endogenous anti-microbial peptide LL-37 in relationship to 25(OH)D, we found that lower levels of 25(OH)D were also associated with lower systemic levels of LL-37. This association supports recent *in vivo *data that vitamin D plays some roles in regulating the production of antimicrobial peptides such as LL-37 in cultured macrophages [3]. Since many cells of the immune system possess the vitamin D receptor, vitamin D status may prove to be an important factor in management of sepsis syndrome and other critical illness.

Vitamin D insufficiency is a common condition in patients admitted to the intensive care unit [5,16-18]. We found that > 95% of our critically ill patients had vitamin D insufficiency. Patients with critical illness likely had vitamin D insufficiency which preceded their hospitalization since several studies have documented a high prevalence of vitamin D insufficiency in hospitalized patients [19-21]. Vitamin D insufficiency continues to remain a health concern in hospitalized patients since few treatment guidelines exist to address vitamin D status. The American Society for Parenteral and Enteral Nutrition recommend only 200 IU of vitamin D daily for hospitalized patients [22]. Heaney estimates that a dose of 400 IU daily would only raise 25(OH)D concentrations by 2.8 ng/mL, leaving most hospitalized patients vitamin D insufficient [23]. Van den Berghe et al evaluated increased vitamin D repletion of critically ill subjects with 500 IU of vitamin D; however, 25(OH)D concentrations still remained in the insufficient range [6]. Thus, these studies suggest that higher recommended doses of vitamin D are likely needed to correct vitamin D insufficiency in hospitalized patients.

We found that vitamin D binding protein (DBP, and also known as Gc-globulin) concentrations were also significantly lower in critically ill subjects with sepsis compared to critically ill subjects without sepsis and healthy control subjects. Our findings are consistent with Dahl et al who reported that lowered DBP was associated with sepsis and organ dysfunction [14]. Vitamin D binding protein is the major carrier protein for circulating 25(OH)D. Adequate levels of DBP are required to recover filtered 25-hydroxyvitamin D lost in the urine [24]. This process is facilitated by megalin, a protein located on the renal epithelial cell which binds to the DBP-25-hydroxyvitamin D complex to facilitate the recovery of filtered vitamin D metabolites [25]. Lower DBP results in further loss of urinary 25(OH)D further exacerbating already low levels of circulating 25(OH)D concentrations.

Vitamin D binding protein not only is a carrier for the two major circulating forms of vitamin D, 25-hydroxyvitamin D and 1,25-dihydroxyvitamin D, but it is also a scavenger of monomeric actin thus preventing its polymerization into F-actin [25,26]. The actin binding characteristics of DBP may play a protective role in sepsis to prevent polymerization of actin released from injured tissue which can in turn result in microembolization of end-organs [25,26]. Actin binding with DBP results in lowered DBP concentrations which in turn further lowers 25(OH)D due to renal wasting of vitamin D and its metabolites, providing another mechanism to explain why vitamin D insufficiency is common in patients with sepsis.

The classic function of vitamin D is to maintain optimal calcium and skeletal homeostasis. Nierman and Mechanick reported the majority of their cohort of chronically ill elderly patients transferred from the intensive care unit had evidence of rapid bone turnover due to vitamin D deficiency [5]. Recent evidence suggests that vitamin D may also play an important role in enhancing innate immunity against infection. Liu et al demonstrated that 1,25(OH)_2_D_3 _treatment of macrophages infected with *Mycobacterium tuberculosis *in vitro resulted in enhanced production of an endogenous anti-microbial peptide, cathelicidin or LL-37, and in improved killing of the microorganisms [3]. LL-37 has a broad antimicrobial spectrum and has been demonstrated to possess multiple other immunoregulatory functions, from chemoattraction of inflammatory cells, to promotion of wound healing, and regulation of angiogenesis [27]. Administration of LL-37 has been demonstrated to be protective in rodent models of sepsis [28,29]. A recent randomized, placebo controlled trial of vitamin D supplementation in patients with pulmonary tuberculosis in Indonesia demonstrated significantly higher sputum conversion rates at earlier time points in the group randomized to receive vitamin D compared to the group assigned placebo [30]. A smaller study of post-menopausal women also suggested that vitamin D may have activity against influenza [31]. Given early findings in pre-clinical studies and some early clinical studies, optimal levels of vitamin D may be necessary for enhanced anti-microbial peptide production for improved innate immunity against infection. No prospective clinical study has confirmed that intervention with vitamin D would raise LL-37 concentrations and improve activity against infection.

One of the potential weaknesses of the study was that the three groups of patients were not equally matched for race which could impact 25(OH)D levels. However, after adjustment for the potential confounder of race, we found that critically ill subjects still had lower vitamin D status than healthy controls. Also, HIV patients were only found in the ICU sepsis group (n = 4, 16.7%). The mean LL-37 of the HIV infected subjects was not statistically different from the overall mean of the sepsis group. HIV patients had significantly lower mean 25(OH)D levels (10.3 ± 5.2 ng/mL); however, due to the small number of HIV patients, it is difficult to ascertain if HIV infection independently influences 25(OH)D concentrations.

Our cross-sectional study design does not allow us to determine whether restoring vitamin D status to optimal levels would increase LL-37 levels systemically or result in improved immunity against infection. It is unknown at this time whether circulating levels of LL-37 translate directly into antimicrobial activity. It is our hypothesis that optimal vitamin D status would translate in increased levels of LL-37 to enhance clearance of infections, but this has yet to be proven in clinical studies. Rigorous intervention-based clinical studies are needed to further delineate the causal relationship between vitamin D and LL-37 in the human host and to assess the clinical implications of this relationship in the setting of critical illness, in particular whether optimization of vitamin D levels are associated with improved clinical outcomes. Also, another limitation of our study is that the acute-phase reaction associated with the medical conditions leading to ICU admission may possibly depress 25(OH)D and LL-37 levels. More clinical studies are needed to examine the effect of vitamin D status and LL-37 on downstream production of inflammatory cytokines and coagulation factors, as these parameters are important in the pathogenesis of sepsis syndrome and other severe illness. Future studies should also focus on whether improved vitamin D status would have a more pronounced effect on levels of LL-37 and other antimicrobial peptides potentially regulated by vitamin D at immunologic barrier sites, such as the surface of the skin [32] and the surface fluid of the respiratory airways [33], in addition to modulating systemic levels of antimicrobial peptides.

In conclusion, we have determined that nearly all critically ill patients we studied had sub-optimal vitamin D status and a higher rate of vitamin D insufficiency compared to healthy subjects. This finding is associated with lower systemic levels of LL-37, a vitamin D dependent antimicrobial peptide which appears to have multiple effector roles within the immune system. Vitamin D binding protein (DBP) levels were also significantly decreased in critically ill subjects with sepsis which further exacerbates vitamin D insufficiency. Whether this effect is due to decreased vitamin D binding protein synthesis, increased clearance and/or increased catabolism is unknown. Vitamin D may have an important role in regulation of the immune system through induction of such antimicrobial peptides in patients with critical illness, who are known to have a high prevalence of vitamin D insufficiency. Results of this clinical study provide important background to perform larger scale, intervention based trials of adjunctive vitamin D therapy in a variety of clinical settings, including further studies in the management of human sepsis syndrome and other critical illnesses.

## Abbreviations list

25(OH)D: is 25-hydroxyvitamin D; 1,25(OH)D: is 1,25-dihydroxyvitamin D; ACCP: is American College of Chest Physicians; ALT: is alanine aminotranferease; AMP: is anti-microbial Peptide; APACHEII: is Acute Physiology and Chronic Health Evaluation II; AST: is aspartate aminotransferase; BUN: is blood urea nitrogen; Cr: is creatinine; DBP: is vitamin D binding protein; DIC: is disseminated intravascular coagulation; ELISA: is Enzyme-Linked ImmunoSorbent Assay; HIV: is human Immunodeficiency Virus; ICU: is intensive care unit; INR: is International Normalized. Ratio; LL-37: is human cathelicidin; M. *Tb*: is *Myobacteria tuberculosis*; PT: is prothrombin time; PTT: is partial thromboplastin time; SCCM: is Society of Critical Care Medicine; SOFA: is sequential organ failure assessment; UVB: is Ultraviolet B; VDR: is vitamin D receptor

## Competing interests

The authors declare that they have no competing interests.

## Authors' contributions

LJ carried out all laboratory studies (immunoassays, sample collection and preparation), helped design the study, drafted the manuscript, organized and carried out initial statistical analysis. AY participated in drafting the manuscript and collecting background information. SJ carried out statistical analysis. HB provided general supervision and was involved in drafting the manuscript. GM provided samples, supervised in study design, and was involved in drafting the manuscript. TZ provided samples, supervised in study design, and was involved in drafting the manuscript. VT carried out the initial conception and design of the study, supervised and assisted in laboratory techniques, and was involved in drafting the manuscript.
